# A Saliva Protein of *Varroa* Mites Contributes to the Toxicity toward *Apis cerana* and the DWV Elevation in *A*. *mellifera*

**DOI:** 10.1038/s41598-018-21736-9

**Published:** 2018-02-21

**Authors:** Yi Zhang, Richou Han

**Affiliations:** Guangdong Public Laboratory of Wild Animal Conservation and Utilization, Guangdong Key Laboratory of Animal Protection and Resource Utilization, Guangdong Institute of Applied Biological Resources, 105 Xingang Road West, Guangzhou, 510260 China

## Abstract

*Varroa destructor* mites express strong avoidance of the *Apis cerana* worker brood in the field. The molecular mechanism for this phenomenon remains unknown. We identified a *Varroa* toxic protein (VTP), which exhibited toxic activity toward *A*. *cerana* worker larvae, in the saliva of these mites, and expressed VTP in an *Escherichia coli* system. We further demonstrated that recombinant VTP killed *A*. *cerana* worker larvae and pupae in the absence of deformed-wing virus (DWV) but was not toxic to *A*. *cerana* worker adults and drones. The recombinant VTP was safe for *A*. *mellifera* individuals, but resulted in elevated DWV titers and the subsequent development of deformed-wing adults. RNAi-mediated suppression of *vtp* gene expression in the mites partially protected *A*. *cerana* larvae. We propose a modified mechanism for *Varroa* mite avoidance of worker brood, due to mutual destruction stress, including the worker larvae blocking *Varroa* mite reproduction and *Varroa* mites killing worker larvae by the saliva toxin. The discovery of VTP should provide a better understanding of *Varroa* pathogenesis, facilitate host-parasite mechanism research and allow the development of effective methods to control these harmful mites.

## Introduction

*Varroa destructor* Anderson & Trueman (Acari: Varroidae) was originally identified as an ectoparasite of the Asian honeybee *Apis cerana*. Before the year 2000, *V*. *destructor* was miscalled *V*. *jacobsoni*. In fact, these two species are different in body shape, cytochrome oxidase (CO-I) gene sequence, and virulence to honey bees^1^. Importantly, *V*. *destructor* can reproduce on *A*. *mellifera* but *V*. *jacobsoni* cannot^1^. *V*. *destructor* was transmitted to the European honeybee *A*. *mellifera* in the 1950s^1,2^ and is now a worldwide ectoparasite of the economically important honeybee, except in Australia^3^. Adult females of this species can be found within sealed drones and worker cells and attached to adult bees^4^. Adult female mites feed on the hemolymph of sealed brood and adult bees^4^. In addition to weakening bee hosts by extracting hemolymph, *V*. *destructor* transmits a number of pathogens to these hosts, notably deformed-wing virus (DWV)^5–8^. Occasionally, DWV may cause deformed-wings in 40% *A*. *mellifera* adults following conditioned artificial infestation^8,9^. The *V*. *destructor* mite has been associated with widespread losses of honeybee colonies in the United States and Europe since 2006^3^, but not in China.

The balanced relationship between the original host *A*. *cerana* and *V*. *destructor* is the most striking aspect of this ectoparasite. Three host factors control the growth of the *Varroa* population and preserve the infested bee colonies as follow: mite infertility in the worker brood^9^, hygienic behaviors^10–13^ and the “entombing” of the drone brood^10,13^. In nature, *Varroa* mites show a marked preference for the drone brood and a strong avoidance of the worker brood in *A*. *cerana* colonies^4,13^. The reason for *Varroa* mites expressing a strong avoidance of the worker brood remains unclear. Recently, we injected crude saliva collected from female *Varroa* mites into *A*. *cerana* and *A*. *mellifera* larvae and observed that this treatment killed *A*. *cerana* worker larvae, while injected *A*. *mellifera* larvae developed into adults with deformed wings. To explore the potential involvement of salivary proteins in the toxicity towards *A*. *cerana* worker larvae, a salivary secretome was established and analyzed (Zhang and Han, unpublished data). Based on the secretome results, we identified a *Varroa* saliva toxic protein (VTP) that exhibited toxic activity towards *A*. *cerana* worker larvae from freshly capped cells in the absence of DWV and increased DWV titers for the development of deformed-wing *A*. *mellifera* adults. We also proposed a modified mechanism for *Varroa* mite avoidance of the worker brood. The discovery of VTP should help elucidate the interaction mechanism between the host and parasite and allow the development of effective anti-mite methods, based on RNAi-mediated and/or gene-editing knockdown of *vtp* receptor genes, for protecting *A*. *mellifera* colonies.

## Results

### Virus contents of the samples

We detected deformed wing virus [DWV] in *A*. *mellifera* larvae, pupae and adult workers and drones, *Varroa* mites and their saliva, and *A*. *cerana* infested with mites or injected with mite saliva but not in *A*. *cerana* that were injected or not injected with E-VTP (E-VTP = the expressed VTP fused with an epitope tag) (Supplementary Table S1). The DWV load in the crude mite saliva was 2.78 × 10^4^ copies/μL. *Nosema ceranae* was present in both bee species (Supplementary Fig. S1).

### Expression of VTP in *Escherichia coli*

**Figure 1:**
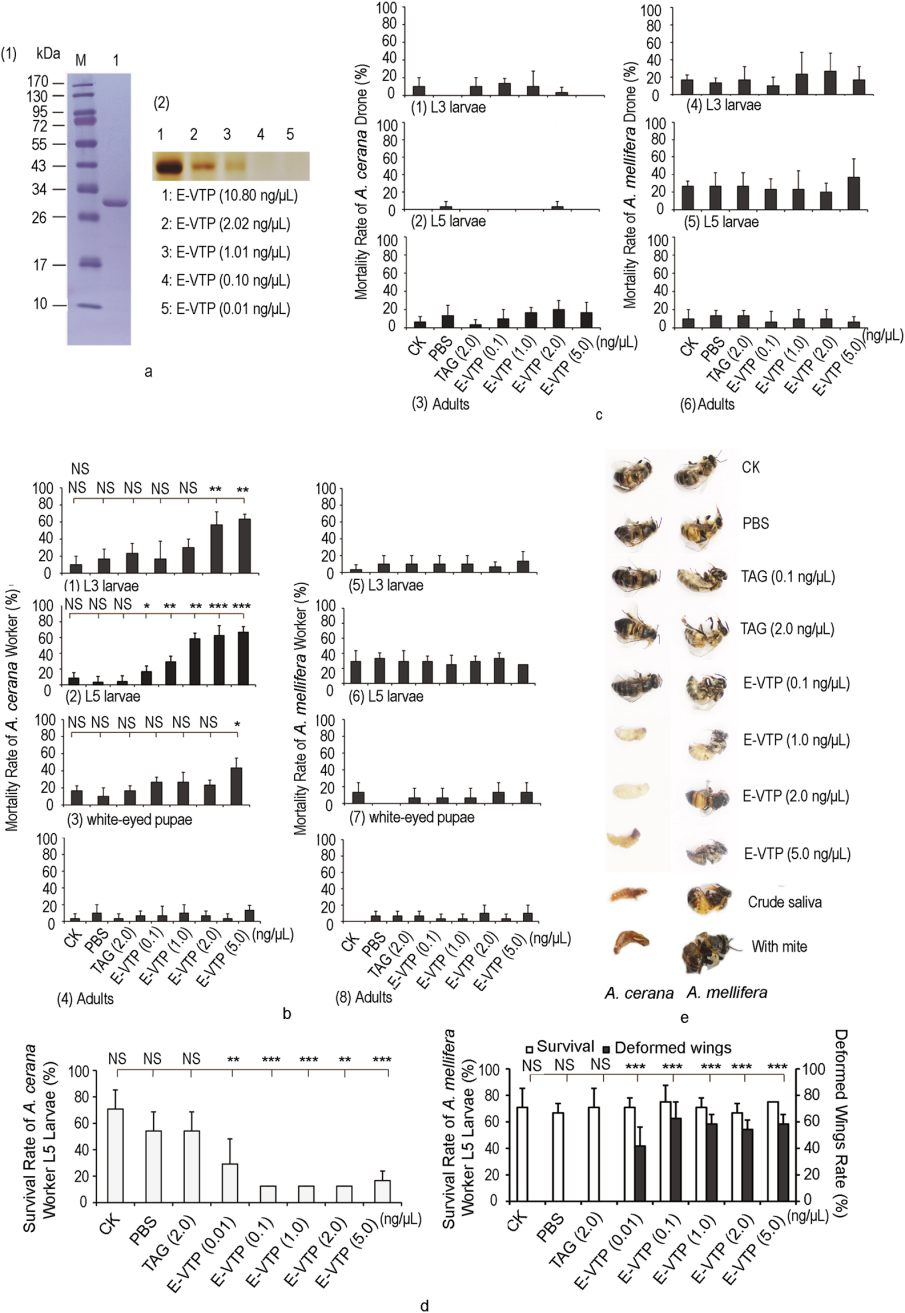
Effects of the purified *Varroa* toxic protein (VTP) expressed in *E*. *coli* on *A*. *cerana* and *A*. *mellifera*. (**a**-(1)) Purified recombinant VTP (lane 1). (**a**-(2)) The purified E-VTP and different dilutions as visualized using silver staining. (**b**) Mortality rates of *A*. *cerana* and *A*. *mellifera* worker L3 larvae, L5 larvae, white-eyed pupae and adults injected with the purified E-VTP at different concentrations (0.1, 1.0, 2.0, and 5.0 ng/μL). CK, L5 larvae not injected; PBS, L5 larvae injected with PBS (10 mM, pH 7.4); TAG, injected with tag protein purified from *E*. *coli* Transetta pET32 (**a**). (**c**) Mortality rates of *A*. *cerana* and *A*. *mellifera* drone L3 larvae, L5 larvae and adults injected with the purified E-VTP at different concentrations (0.1, 1.0, 2.0, and 5.0 ng/μL). (**d**) Emergence rates and deformed wings rates of *A*. *mellifera* worker L5 larvae injected with the purified E-VTP at different concentrations (0.1, 1.0, 2.0, and 5.0 ng/μL). (**e**) Morphology of *A*. *cerana* and *A*. *mellifera* developed from worker L5 larvae with different treatments. One-way ANOVA with post hoc multiple comparison tests, NS, not significant; *P < 0.05, ***P < 0.001, n = 8 for L5 larvae, n = 10 for white-eyed pupae and adults, in each replicate per treatment. Three replicates were established. The data are presented as the mean values ± S.D.

The *vtp* cDNA sequence contains 405 bp and encodes 135 amino acids with a predicted signal peptide at the site between aa 16 and 17 (Supplementary Fig. S2); the predicted isoelectric point (PI) of VTP is 5.27 and its predicted molecular weight (MW) is 14.66 kDa. VTP has no homologs with known protein families and contains neither known domains nor repeated sequences, except a 29% homology to neuroligin-4 (Y-linked-like, XP_018496732) from *Metaseiulus occidentalis*. VTP possesses 4–6 α-helices in its secondary structure. The *vtp* cDNA sequence was submitted to the GenBank database under accession number KU647280. We also amplified the *vtp* DNA sequence using *V*. *destructor* genomic DNA as a template. The full-length *vtp* sequence contained 952 bp and included 3 introns (bp 79–219, 271–542, and 627–760), which confirmed the survey sequence of the *Varroa* genome^14^. We purified recombinant VTP from *Escherichia coli* Transetta (DE3) cells. The fusion protein (E-VTP) was estimated to have a MW of ~34.8 kDa (Fig. 1a), which was confirmed by Western blotting analysis (Supplementary Fig. S3).

### Effects of purified E-VTP on honeybees

We found that the mortality rates of *A*. *cerana* worker larvae and pupae challenged with purified E-VTP were significantly higher than those of the controls and increased with the concentrations of injected E-VTP at 2 days (Fig. 1b-(1), L3 larvae: df = 6, 14, F = 5.194, *P* = 0.005; (2), L5 larvae: df = 7, 16, F = 15.644, *P* = 0.000; (3), pupae: df = 6, 14, F = 3.818, *P* = 0.018). No DWV was detected from these worker larvae and pupae, even after injecting purified E-VTP (Supplementary Table S1). The cadavers also exhibited the same traits as those resulting from infesting *A*. *cerana* worker L5 larvae with mites or injecting these insects with crude mite saliva (Fig. 1e). The survival rates of *A*. *cerana* worker L5 larvae injected with E-VTP were significantly lower than those of the controls (Fig. 1d, df = 7, 16, F = 96.533, *P* = 0.000). No deformed wings were found in *A*. *cerana* worker adults.

When *A*. *mellifera* worker larvae and pupae were injected with purified E-VTP at different concentrations, we found that their mortality rates were similar to those of the controls at 2 days (Fig. 1b-(5), L3 larvae: df = 6, 14, F = 0.238, *P* = 0.956; (6), L5 larvae: df = 7, 16, F = 0.295, *P* = 0.946; (7): pupae: df = 6, 14, F = 1.653, *P* = 0.205); the survival rates of the worker larvae and pupae were not significantly different among the treatments and the controls. However, 40–60% of the resulting adults from the injected worker larvae had deformed wings (Fig. 1d). The DWV loads of *A*. *mellifera* wing-deformed adults were significantly higher than those of normal-winged adults (Supplementary Fig. S4), indicating that the E-VTP injection of *A*. *mellifera* worker L5 larvae significantly increased the DWV titers in the resulting adults. Similar wing traits were found in the adults after *A*. *mellifera* worker L5 larvae were challenged with E-VTP or with mites or injected with crude mite saliva (Fig. 1e). When the control materials were injected into *A*. *mellifera* worker L5 larvae, none of the resulting adults had increasing DWV loads or deformed wings. These results indicated that introducing VTP into *A*. *mellifera* worker larvae stimulated DWV proliferation and that high DWV titers caused deformed wing adults.

When we injected E-VTP into *A*. *cerana* and *A*. *mellifera* worker adults (Fig. 1b-(4), df = 8, 18, F = 0.598, *P* = 0.767; (8), df = 8, 18, F = 0.668, *P* = 0.713), no significant difference in their mortalities was observed.

The mortality rates of the drone larvae (L3 and L5 larvae) of both bee species (Fig. 1c-(1), df = 6, 14, F = 0.833, *P* = 0.564; (2), df = 6, 14, F = 1.562, *P* = 0.230; (3), df = 6, 14, F = 1.362, *P* = 0.295; (4), df = 6, 14, F = 0.357, *P* = 0.894; (5), df = 6, 14, F = 0.293, *P* = 0.930; (6), df = 6, 14, F = 0.439, *P* = 0.841) were not significantly different among the treatments and the controls, indicating that E-VTP was not toxic to the drone larvae. The worker and drone adults of the two bee species were alive for at least 7 days under laboratory conditions.

### RNAi of *vtp* gene in the mites

**Figure 2:**
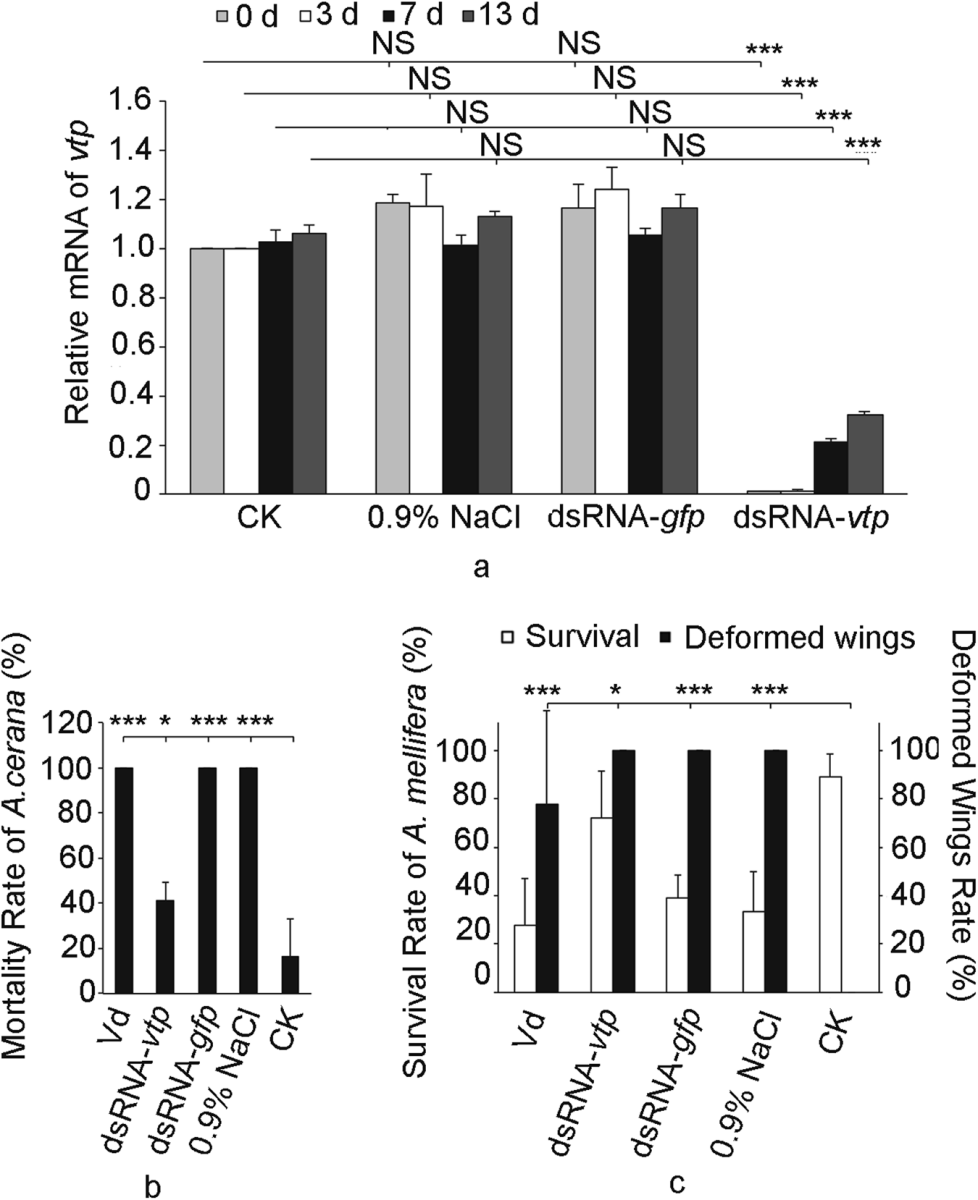
RNAi-mediated knock down of *vtp* expression in adult *V*. *destructor* and its effects on toxicity toward honeybees. Eight live mites were collected from each treatment group, for 2 repeats. The mites were treated by overnight (approximately 15 hours) immersion in different solutions at 16 °C and subsequently placed on *A*. *mellifera* and *A*. *cerana* worker L5 larvae. (**a**) CK, *V*. *destructor* mites that were not treated; 0.9% NaCl, mites treated with 0.9% NaCl; dsRNA-*gfp*, mites treated with dsRNA-*gfp* (2.5 μg/μL); dsRNA-*vtp*, mites treated with dsRNA-*vtp* (2.5 μg/μL). **0 d**, mites collected immediately after immersion; **3 d**, treated mites placed on L5 larvae for 3 days; **7 d**, treated mites placed on L5 larvae for 7 days; **13 d**, treated mites placed on L5 larvae for 13 days, when eclosion was observed. One-way ANOVA with post hoc multiple comparison tests, NS, not significant; ***P < 0.001, the values were expressed as the mean values ± S.D. (**b**) Mortality rate of *A*. *cerana* worker L5 larvae infested by *Varroa* mites; (**c**) Percentage of adult eclosion and percentage of eclosed adults with deformed wings resulting from *A*. *mellifera* worker L5 larvae infested by *Varroa* mites. CK, worker L5 larvae without mite infestation; *Vd*, worker L5 larvae exposed to *Varroa* mites that were not treated; 0.9% NaCl, worker L5 larvae exposed to *Varroa* mites treated with 0.9% NaCl; dsRNA-*gfp*, worker L5 larvae exposed to *Varroa* mites treated with dsRNA-*gfp* (2.5 μg/μL); dsRNA-*vtp*, worker L5 larvae exposed to *Varroa* mites treated with dsRNA-*vtp* (2.5 μg/μL). One-way ANOVA with post hoc multiple comparison tests, *P < 0.05, ***P < 0.001, n = 6 L5 larvae in each of three replicates per treatment. The data are presented as the mean values ± S.D. The experiment was repeated more than two times.

We detected very low levels of *vtp* mRNA in dsRNA*-vtp*-treated mites on days 0, 3, 7 and 13 post-treatment but observed normal *vtp* mRNA levels in mites treated with 0.9% saline or dsRNA-*gfp* (Fig. 2a, 0d, df = 3, 8, F = 387.725, *P* = 0.000; 3d, df = 3, 8, F = 160.393, *P* = 0.000; 7d, df = 3, 8, F = 4514.652, *P* = 0.000; 13d, df = 3, 8, F = 765.466, *P* = 0.000). Interestingly, we found that the mortality rate of *A*. *cerana* worker L5 larvae infested with mites with a low *vtp* mRNA level was 41.1% of that of the controls, suggesting that the RNAi-mediated knock down of *vtp* gene expression protected *A*. *cerana* worker L5 larvae from the effects of *V*. *destructor* mites (Fig. 2b, df = 4, 10, F = 48.903, *P* = 0.000). Given the same treatment, the survival rate of *Varroa*-susceptible *A*. *mellifera* worker L5 larvae was significantly increased, but the resulting adults had high DWV titers (Supplementary Fig. S5) and deformed wings (Fig. 2c, df = 4, 10, F = 22.866, *P* = 0.000), which is probably due to the increasing *vtp* gene expression in the RNAi-treated mites after 13 days (Fig. 2a).

## Discussion

The co-adaptation of *V*. *destructor* and *A*. *cerana* results from an interesting dynamic balance between the host and parasite populations and accounts for the resistance of *A*. *cerana* to these mites. Effective grooming and hygienic behavior, non-production of the mites in the worker brood, and the “entombing” of the infested drone brood are reportedly the major factors ensuring this resistance^10–13,15,16^. Grooming and hygienic behavior reduces the susceptibility of *A*. *cerana* colony to *V*. *destructor* infestation^16–19^ but seems to be highly variable^20^. The higher susceptibility of mite-infested worker brood of *A*. *cerana* leads to more efficient hygienic behavior^21^. However, the removal of the *Varroa*-infested worker brood does not necessarily induce the death of the mites, as most mites may escape from brood cells that were opened during removal^10^. Interestingly, *V*. *destructor* mites or the injection of mite saliva can kill *A*. *cerana* worker larvae^21,22^. *A*. *cerana* larvae block *Varroa* mite reproduction in the infected worker brood^9^ and are sacrificed due to mite saliva toxin (similar to a mutual destruction strategy). Lack of production of mites in the worker brood was not caused by the death of *A*. *cerana* larvae, as these mites, fed with fresh *A*. *cerana* or *A*. *mellifera* larvae, were also infertile (unpublished results). The reason why these mites produce a toxic protein to kill *A*. *cerana* larvae remains unknown. However, in the field, *V*. *destructor* mites exhibit a marked preference for the drone brood and are rarely found in the worker brood of *A*. *cerana* colonies as long as a sufficient drone brood is available^13^. This phenomenon is considered crucial for the balanced host-parasite relationship in *A*. *cerana* during the long co-evolution of these species^13^. Based on the results of the present study, we suggest a mechanism to explain why *Varroa* mites express the strong avoidance of the worker brood. Under mutual destruction stress, *Varroa* mites likely evolved to protect themselves in the field by avoiding the worker brood, instead by preferring drone brood where they do not kill drones by the saliva toxin and subsequently reproduce. This strategy significantly reduces mite death and non-reproduction risks. The mite infestation of drone brood typically does not induce a significantly negative effect on colony size^23^, therefore almost the entire *A*. *cerana* colony can be protected. Thus, we propose a modified mechanism for the mite avoidance of the worker brood, which is probably due to mutual destruction stress.

In contrast to *A*. *cerana* worker larvae, infected *A*. *mellifera* workers and drones support the fertility of *V*. *destructor* mites, and VTP is neither toxic to the workers nor to the drones of *A*. *mellifera*. This finding indicates that *A*. *mellifera* individuals are less susceptible to this mite. However, social behaviors, such as grooming, and hygienic behaviors in the *A*. *mellifera* colony were much weaker than those in the *A*. *cerana* colony^4^, resulting in damage to the *A*. *mellifera* colony by the mites. Furthermore, two unique tolerance factors in the *A*. *cerana* colony, non-reproduction in the worker brood and entombing of drone larvae, do not occur in *A*. *mellifera*. These findings imply that *A*. *mellifera* colony is more susceptible to this mite than *A*. *cerana*, the original host of this mite. It is reasonable to imagine that without strong avoidance of the worker brood and the toxic effect on *A*. *mellifera* larvae, *V*. *destructor* successfully parasitizes the worker brood in *A*. *mellifera*. However, why *A*. *mellifera* individuals are less susceptible to VTP is unclear. This VTP contains neither known domains nor repeated sequences, but 4–6 α-helices in its secondary structure. The function of VTP receptors and/or detoxification factors in *A*. *mellifera* hemolymph deserves further research.

Insects are generally attacked by many types of parasites. These parasites may secrete venomous proteins and non-proteinaceous compounds^24–28^ that alter the immunity and/or modulate the development and physiology of the hosts or, in few cases, kill the hosts^28^. However, these proteins or compounds do not directly kill the hosts, and knowledge of the characteristics of individual salivary components is limited^28^. Unexpectedly, *V*. *destructor* mites deliver a toxic protein from the saliva to kill the infected host, which in turn prevents the reproduction of these mites in worker broods. This mutual destruction strategy may be too costly for the mites and *A*. *cerana* bees. However, from the point of view of evolutionary pressure, we can explain why these mites are rarely found in the worker brood cells of *A*. *cerana* colonies in the field^4^. VTP is not toxic to the drone of *A*. *cerana*, potentially explaining why the reproduction of *Varroa* mites in the original host *A*. *cerana* is limited to drone brood, although the reasons why the drones are resistant to the *Varroa* mites deserve further research. VTP is also safe to the worker adults of both bee species, which may reflect the importance of evolutionary adaptation, as the mites need the worker adults for transportation into other colonies^29^, and the bee colony may be collapsed if the infected worker adults could be killed by the mites.

DWV is prevalent in *A*. *mellifera* and *V*. *destructor* but is not common in *A*. *cerana*^30–32^. Although the introduction of higher titers of recombinant DWV into bee pupae caused *A*. *mellifera* adult wing deformities^33^, in absence of *V*. *destructor* infestation or with lower titers of DWV in the mite-infested bees^33^, *A*. *mellifera* adults did not show wing-deformities^6^. Interestingly, *Varroa* mites have been feeding on European bees on an isolated Brazilian island for decades, but do not activate DWV or cause deformed bees or colony losses^34^. The reasons for these results remain unknown. The following factors should be considered: the bees are not sensitive to DWV infection; the mites may lose or contain less toxin protein; DWV is a weakly pathogenic isolate. In the present study, although *A*. *cerana* adults from E-VTP-injected larvae showed no deformities wings, we found that introducing E-VTP into *A*. *mellifera* larvae increased DWV loads in the adults, which subsequently developed the deformed-wing trait. Although VTP together with DWV did not generally cause the death of the larvae of normal *A*. *mellifera* colonies, we observed that infestation with *Varroa* mites decreased the emergence rate of a *Varroa*-sensitive *A*. *mellifera* colony in presence of DWV. The RNAi-mediated suppression of *vtp* gene expression in the mites significantly increased the emergence rate of the larvae in this *A*. *mellifera* colony. Thus, these results would promote the development of anti-mite products for the protection of *A*. *mellifera* colonies, based on RNAi-mediated and/or gene-editing (such as CRISPR/cas9) knockdown of *vtp* receptor(s) in *A*. *mellifera*.

## Methods and Materials

### Mites and insects

Using a camel hair brush, mature female *V*. *destructor* mites were collected from the worker pupae in *A*. *mellifera* hives that had not been treated with acaricides in an apiary in Conghua, Guangzhou, China. These mites were placed in sterile Petri dishes (diameter = 9 cm; 20 mites per dish) and used for bioassays within one hour.

The 3^rd^ stage larvae (L3 larvae) and 5^th^ stage larvae (L5 larvae; spinning phase) from freshly capped cells, and the pupae (white-eyed, unpigmented cuticle pupae) and adults of drones and workers of *A*. *mellifera* or *A*. *cerana* derived from apiaries without chalkbrood and foulbrood symptoms in Conghua, Guangzhou were collected according to Kanbar and Engels^35^ and used for bioassays in the laboratory^36^. The developmental stages of the honeybees were defined according to Bitondi *et al*.^37,38^.

### Viral and microsporidia loads

We used RT-PCR assays^30–32,39–49^ to examine the mites and mite saliva collected according to Richards *et al*.^24^. The honeybees used in the present study were examined for the presence of 16 bee viruses using available sequences from NCBI (acute bee paralysis virus [ABPV], chronic bee paralysis virus [CBPV], deformed wing virus [DWV], Kashmir bee virus [KBV], sacbrood virus [SBV] and Israeli acute paralysis virus [IAPV], black queen cell virus [BQCV], *A*. *mellifera* filamentous virus [AmFV], Big Sioux River virus [BSRV], Lake Sinai virus complex [LSV], slow bee paralysis virus [SBPV], *V*. *destructor* macula-like virus [VdMLV]), cloudy wing virus [CWV], Apis iridescent virus [AIV], *Varroa destructor* virus 1 [VDV-1], Moku virus [MV], and two microsporidia parasites (*Nosema apis* and *N*. *ceranae*). Total RNA was isolated from honey bees (larvae, pupae and adults of workers and drones), mite saliva and mites using RNAqueous kits (Ambion, Austin, TX, USA) according to the manufacturer’s instructions. The isolated RNA was quantified by spectrophotometry. DNA was removed from the samples by incubation with DNase I (5 units of DNase I in an appropriate buffer containing the RNase inhibitor RNAsin [Invitrogen, Carlsbad, CA, USA]) at 37 °C for 45 min. The primers for detecting each of the 16 viruses and two microsporidia parasites (Supplementary Table S2) were selected based on previous reports^31,32,39–49^. The cDNA synthesis was performed using a PrimeScript^TM^ 1st Strand cDNA Synthesis Kit (TaKaRa, Dalian, China). The PCR amplification program involved an initial denaturation cycle at 95 °C for 5 min, followed by 35 cycles at 95 °C for 30 s, 55 °C for 30 s, and 72 °C for 1 min, with a final cycle at 72 °C for 7 min. The PCR products were sequenced using the corresponding specific primers.

### Amplification of the full-length sequence of the *vtp* gene

We obtained the partial amino acid sequence of a metalloprotease homolog (herein called VTP) based on the *Varroa* mite salivary secretome (Zhang and Han, unpublished data). To obtain the full-length sequence of the *vtp* gene, we isolated total RNA from pooled samples of adult females *Varroa* mites using RNAiso Plus reagent (No. 9109, TaKaRa, Dalian, China). The full *vtp* cDNA was cloned using the SMARTer™ RACE cDNA Amplification Kit (No. 634924, Clontech, TaKaRa, Tokyo. Japan) according to the manufacturer’s instructions. For 5′-cDNA amplification, the R primer 5′-TCCATCRTGHKKWSMACC-3′ and the UPM primer (5′-CTAATACGACTCACTATAGGGCAAGCAGTGGTATCAACGCAGAGT-3′) from the kit were used. The following PCR program was used: 95 °C for 5 min, followed by 35 cycles at 94 °C for 30 s, 45 °C for 30 s, and 72 °C for 45 s, with a final cycle at 72 °C for 10 min. For 3′-cDNA amplification, the forward primer 5’- ATGTCGGGACTCAGCCTGAAATTGTGGAT-3′ was designed, and touchdown PCR was performed using Titanium Taq DNA Polymerase (No. 639206, Clontech, TaKaRa, Tokyo, Japan). The PCR program contained 5 cycles at 94 °C for 30 s and 72 °C for 1.5 min; 5 cycles at 94 °C for 30 s, 65 °C for 30 s and 72 °C for 1.5 min; and 25 cycles at 94 °C for 30 s, 55 °C for 30 s, and 72 °C for 1.5 min. The PCR products were visualized on a 1.1% agarose gel and subsequently purified and cloned into the T-vector for sequencing.

To determine the full-length sequence of the *vtp* gene, we also isolated genomic DNA from six sterilized *V*. *destructor* eggs (sterilized using 70% ethanol) using the KAPA Express Extraction Kit (No. KK7101, Kapa Biosystems, Boston, USA). The *vtp* gene was PCR amplified from genomic DNA using PfuUltra II Fusion HS DNA Polymerase (Stratagene, Heidelberg, Germany) and the primers VTP-F-EcoRI (5′-*GAATTC***ATG**TTCAAACTTCTCGTTATCG-3′) (*EcoR*I) and VTP-R-HindIII (5′-*AAGCTT***TTA**GGAGGCGAGCGCCTGCTGGA-3′) (*Hind*III). The PCR product was purified and cloned into the pEASY-Blunt cloning vector (Transgen BioTech, Beijing, China), after which the resulting plasmid was transformed into *E*. *coli* Trans T1 (Transgen BioTech, Beijing, China). The start codon (ATG) and stop codon (TAA) in the primers are indicated in bold, and the restriction sites are indicated in italics. Two clones were sent to Invitrogen LTD. (Shanghai) for DNA sequencing. The resulting sequence was analyzed using DNASTAR software and the ClustalW program and was submitted to GenBank under accession number (KU647280). The translated protein sequence was analyzed by Expasy (http://web.expasy.org/protparam/). The signal peptide, secondary structure and subcellular localization of the translated protein were predicted by the SignalP 4.1 Server (http://www.cbs.dtu.dk/services/SignalP), Interpro (http://www.ebi.ac.uk/interpro/sea-rch/sequence-search) and TargetP (http://www.cbs.dtu.dk/services/TargetP/), respectively.

### Western blotting analysis

To confirm the VTP protein in the saliva of the mites, we prepared mite saliva, purified E-VTP and TAG protein as described above and analyzed these proteins via Western blotting. The proteins were transferred to a polyvinylidene difluoride (PVDF) membrane. The membrane was incubated overnight at 4 °C with primary rabbit polyclonal anti-VTP antibodies (diluted 1:1000) produced by AB Clonal Technology (Yingji Biotechnology, Shanghai, China). Following three washes, the membranes were incubated with horseradish peroxidase-conjugated goat anti-rabbit secondary antibody (Boster Biological Technology, Wuhan, China) at a 1:1000 dilution for 1 hour. The immunoreactive protein bands were detected using DAB Western Blotting substrate (Boster Biological Technology, Wuhan, China).

### Expression of VTP in *Escherichia coli* and bee bioassay

We cloned the amplified gene cDNA into the p*EASY*-T1-Simple vector and transformed the resulting plasmid into *E*. *coli* TransT1 cells (Transgen BioTech, Beijing, China). DNA sequencing was performed by Invitrogen (Shanghai, China). The p*EASY*-T1-Simple-*vtp* plasmid was digested using *EcoR*I and *Hind*III. The resulting *vtp* fragment was purified and subsequently ligated into the expression vector pET-32a(+) (Novagen, Darmstadt, Germany), which has an N-terminal His-Tag/thrombin/S-Tag enterokinase configuration plus an optional C-terminal His-Tag sequence. This plasmid was digested using *EcoR*I and *Hind*III to yield pET-32a(+)-*vtp*.

The resulting plasmid was transformed into *E*. *coli* Transetta cells (Transgen BioTech, Beijing, China) to create Transetta/pET-32a(+)-*vtp* for expression of the recombinant protein VTP (=E-VTP). The vector without insert sequence was transformed into the same *E*. *coli* strain to express the tag protein (approximately 20.2 kDa) as a control (=TAG). A single bacterial colony was grown overnight in 15 mL of LB-carbenicillin (100 μg/mL) broth. The culture was diluted 3:100 in 200 mL of fresh LB-carbenicillin medium and was grown until the OD_600_ value reached 0.6. For E-VTP induction, isopropyl-beta-D-thiogalactopyranoside (IPTG) was added at a final concentration of 0.2 mM at 16 °C overnight. E-VTP was isolated from the soluble protein fraction using a Bio-Scale Mini Profinity IMAC cartridge (No. 7324610, Bio-Rad, Berkely, CA, USA) according to the manufacturer’s instructions. The purity of the obtained protein was evaluated using SDS-PAGE, and the protein concentration was determined by using the Modified Bradford Protein Assay Kit (Sangon, Shanghai, China).

A 0.2-μL aliquot of the purified E-VTP at different concentrations (0.10, 1.0, 2.0, and 5.0 ng/μL) was injected into *A*. *mellifera* and *A*. *cerana* L3 larvae (L3 larvae are never naturally fed on by *Varroa*, and this is only an artificial situation), L5 larvae, pupae and adults of workers and drones, using a microinjector (IM-31, Narishige, Tokyo, Japan). Larvae and pupae were injected into the hemocoel of each insect near the end of the abdomen. The bled larvae were discarded after injection. The adults of workers were anesthetized using nitrogen for 8 min prior to injection^50^. After injection, the larvae were reared with 30 μL of larval food^51^ and the adults were fed 3 mL of sugar solution (w/v, 50%) in a culture plate in a growth cabinet at 34 °C and 80% RH. Fresh food was provided to the larvae at 12-h intervals. After 60 hours, the larvae were transferred to a culture plate sealed with Parafilm. Various negative controls, such as uninjected bees (=CK) and bees injected with only PBS (=PBS) or only the TAG protein, were established, with 3 replicates per treatment. The L5 larvae were also injected with crude saliva as a positive control. The bees were assessed daily for mortality, emergence or deformed wing rate (for *A*. *mellifera*).

### RNAi-mediated knock down of *vtp* gene expression

To determine the functions of VTP, we knocked down the expression of *vtp* using the RNAi method^52^. Briefly, dsRNA-*vtp* was prepared using the MEGAscript kit (AM1334, Ambion, Carlsbad, CA, USA) according to the manufacturer’s instructions. The dsRNA-*gfp* was prepared for use as a negative control. A 405-bp (bases 1–405) fragment of the *vtp* gene was amplified from the cDNA transcripts of a female *V*. *destructor* using *vtp*-specific primers (VTP-F/R), and the GFP coding region was amplified from the control GFP-plasmid using GFP-specific primers (GFP-F, TCAAGAAGGACCATGTGGTC and GFP-R, TTCCATGGCCAACACTTGTCC). The dsRNAs were generated using the Ambion MEGAscript T7 Kit and subsequently purified using MEGAclear (AM 1908, Ambion, Carlsbad, CA, USA). The resulting dsRNAs were ethanol-precipitated, resuspended in 0.9% NaCl at a working concentration of 2.5 μg/μL^52^, and subsequently stored at −80 °C until further use. Adult mites collected from capped brood cells were soaked overnight in 500-μL microfuge tubes containing 15 μL of dsRNA-*vtp*, dsRNA-*gfp* (both at 2.5 μg/μL) or 0.9% NaCl at 16 °C, and subsequently transferred to cell culture plates containing L5 larvae of *A*. *mellifera* or *A*. *cerana*. A *Varroa*-sensitive *A*. *mellifera* population from Gaozhou, Guangdong was employed to determine the effect of the RNAi-mediated knock down of *vtp* expression on the mortality of *A*. *mellifera* L5 larvae. Two mites and one L5 larva were placed in each culture plate, and the plates were maintained at 33 ± 1 °C and 80% RH in a growth cabinet (SANYO, Tokyo, Japan) for 12 days until the adults emerged^52^. Emerged adults with normal or deformed wings were frozen using liquid N2 and stored at −80 °C for subsequent RNA extraction to determine their DWV titers^32,33^.

We also employed qRT-PCR to determine the effect of knocking down the expression of *vtp* using RNAi. The total RNA was extracted from mites at 0, 3, 7 and 13 d after the various treatments described above to evaluate the persistence of the effect of *vtp* RNAi. After DNase I treatment, the RNAs were reverse-transcribed using a TransScript All-in-One First-Strand cDNA Synthesis SuperMix for PCR Kit (Transgen BioTech, Beijing, China). The PCR reactions were conducted in triplicate in an Mx3000P Real-Time PCR System (Stratagene, California, USA) using SYBR Green (SYBR Premix Ex Taq II [Tli RNaseH Plus], TaKaRa, Dalian, China). A control without template was included in all PCR batches. The *vtp* primer pairs used were VTP-qF (AACGCATTCAAGACTACATCACCAA) and VTP-qR (CTTTGACAACGTTCTCCTTCTGCT). The *V*. *destructor actin* gene was used for normalization and amplified using *Actin*-F (CATCACCATTGGTAACGAG) and *Actin*-R (CGATCCAGACGGAATACTT) primers. The PCR program included a single cycle at 95 °C for 5 min and 40 cycles at 95 °C for 15 s, 58 °C for 30 s and 72 °C for 30 s. To obtain dissociation curves, the PCR products were heated to 95 °C for 15 s, cooled to 58 °C for 60 s and subsequently heated to 95 °C for 15 s. The mRNA levels relative to that of *actin* mRNA were calculated using Mx3000P Software (version 4.1) (Agilent, Palo Aito, CA, USA). The fold-differences in the mRNA levels were calculated using the 2^−∆∆Ct^ method.

### Data analysis

The data were analyzed using a normal one-way analysis of variance (ANOVA) using SPSS statistical software (16.0), and the significance of the between-treatment differences was evaluated using Duncan’s multiple range test, with significance indicated at a *P* value of <0.05. All data were expressed as percentages and arcsine-square root transformed prior to conducting ANOVA. The values are expressed as the mean values ± S.D. A *P* value of <0.05 indicated statistical significance.

## Electronic supplementary material

Supplementary information
