# Supplementary material for: A Saliva Protein of Varroa Mites Contributes to the Toxicity toward Apis cerana and the DWV Elevation in A. mellifera
Source: Sci Rep. 2018 Feb 21;8:3387. doi: 10.1038/s41598-018-21736-9 (PMC5821841; doi:10.1038/s41598-018-21736-9)
Supplement: Supplementary file 1 — Supplementary information [file 41598_2018_21736_MOESM1_ESM.pdf]

Supplementary information-SREP-17-35449B

A Saliva Protein of *Varroa* Mites Contributes to the Toxicity toward *Apis cerana* and the DWV Elevation in *A. mellifera*

Yi Zhang, Richou Han*

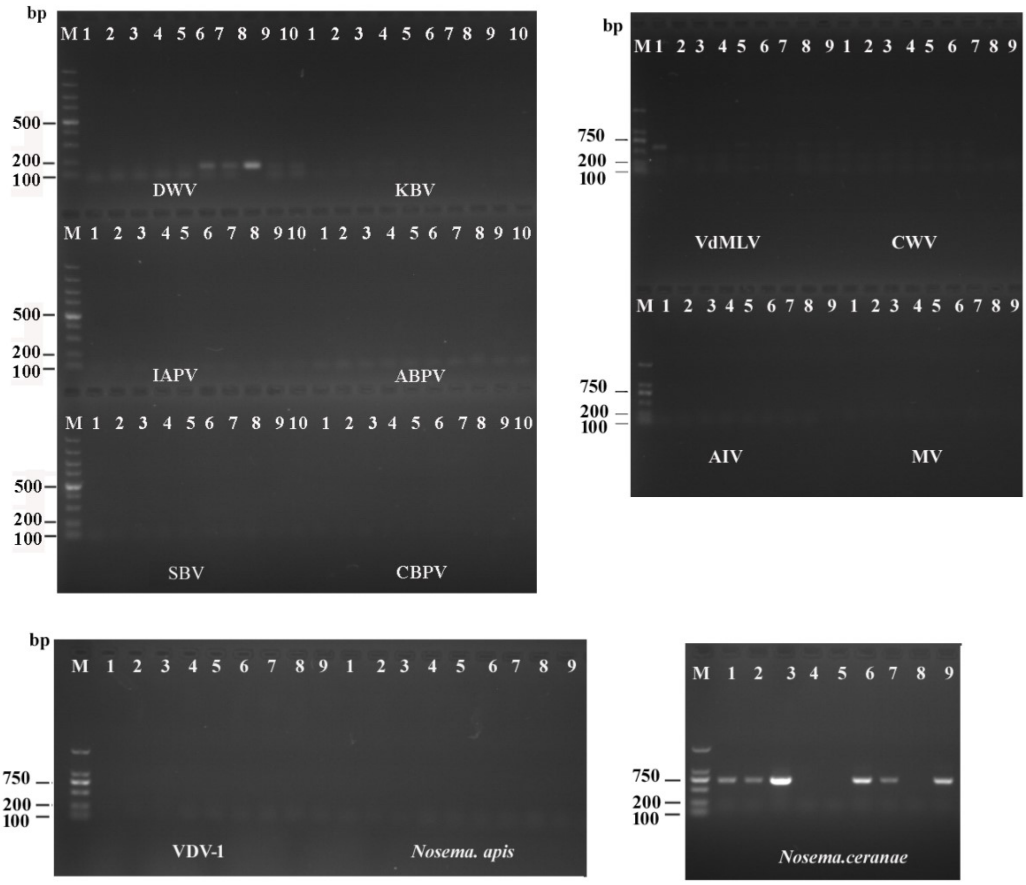

Supplementary Figure S1. Detection of viruses and *Nosema* from worker L5 larvae of *A. mellifera* after VTP injection.1, 9: L5 worker larvae without injection; 2: L5 worker larvae injected with PBS (10 mM, pH 7.4); 3, 8: L5 worker larvae injected with tag protein purified from *E. coli* Transetta pET32(a); 4, 5, 6, 7, 8: the adults developed from worker L5 larvae injected with the purified E-VTP at different concentrations (0.01 ng/μL, 0.1 ng/μL, 1.0 ng/μL, 2.0 ng/μL, and 5.0 ng/μL).

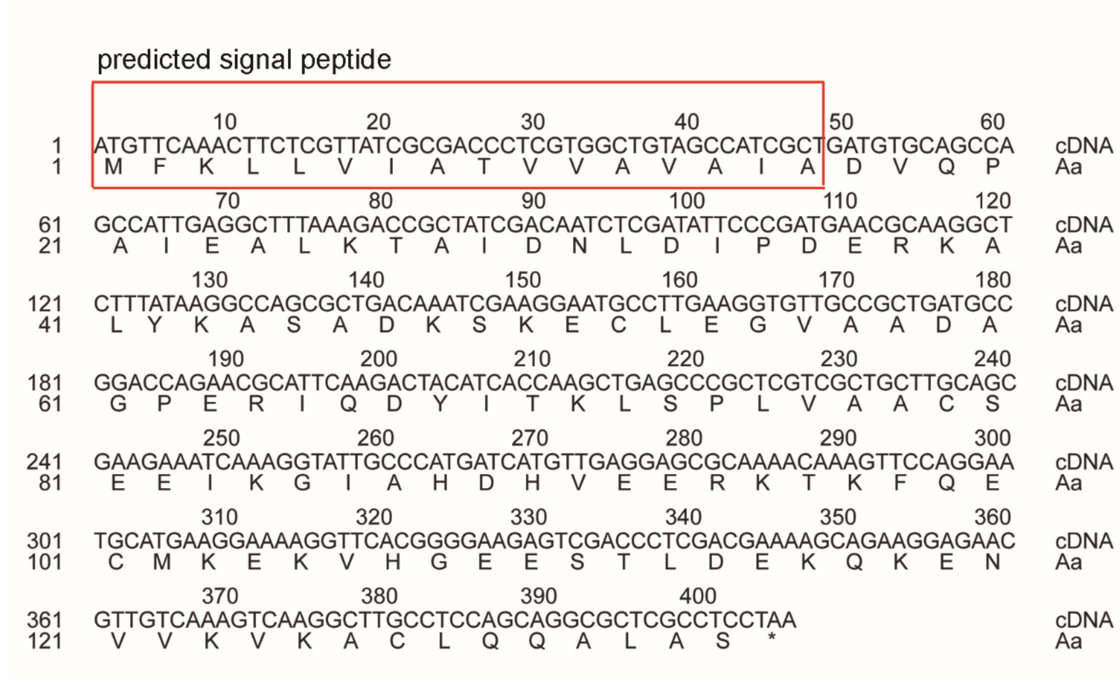

Supplementary Figure S2. The cDNA and amino acid sequences of the *Varroa* toxic protein (VTP).

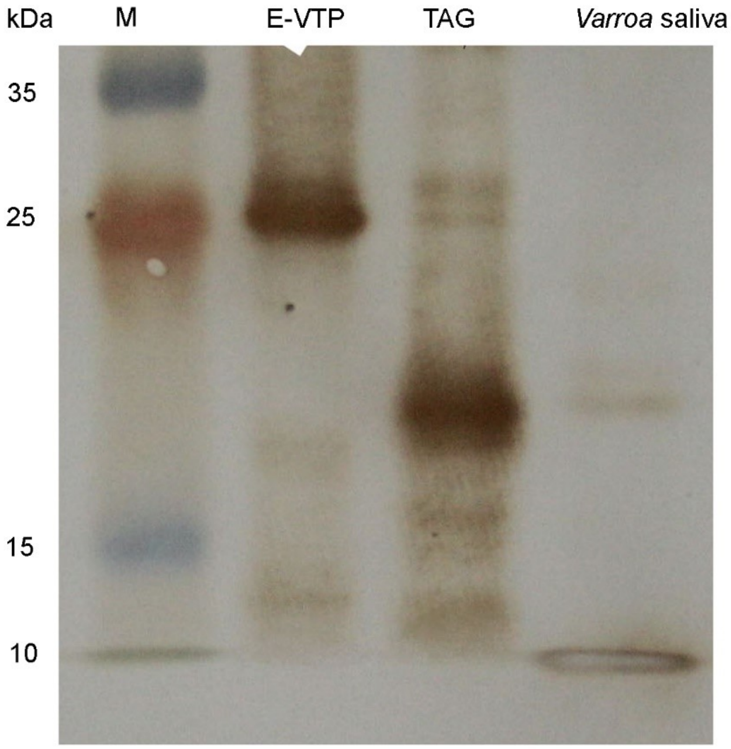

Supplementary Figure S3. Western blot analysis of VTP in crude *Varroa* saliva. M, Pre-stained protein marker; *Varroa* saliva: crude saliva of *V. destructor*; E-VTP, purified recombinant fusion VTP; TAG, purified tag protein.

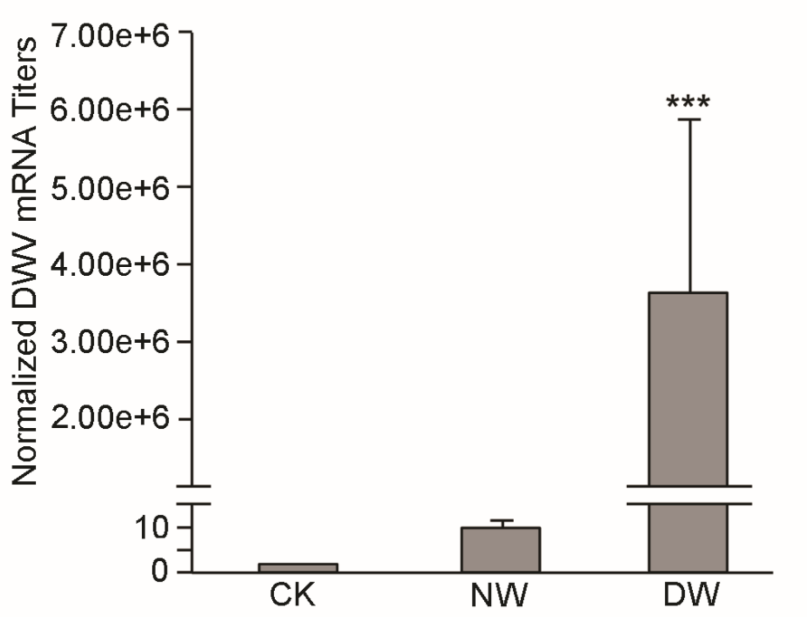

Supplementary Figure S4. Quantitative titers of DWV in *A. mellifera* adults. CK: Adults with normal wings and without injection at L5 larvae; NW: Adults with normal wings and PBS injection at L5 larvae; DW: Adults with deformed wings after injected with E-VTP at L5 larvae.

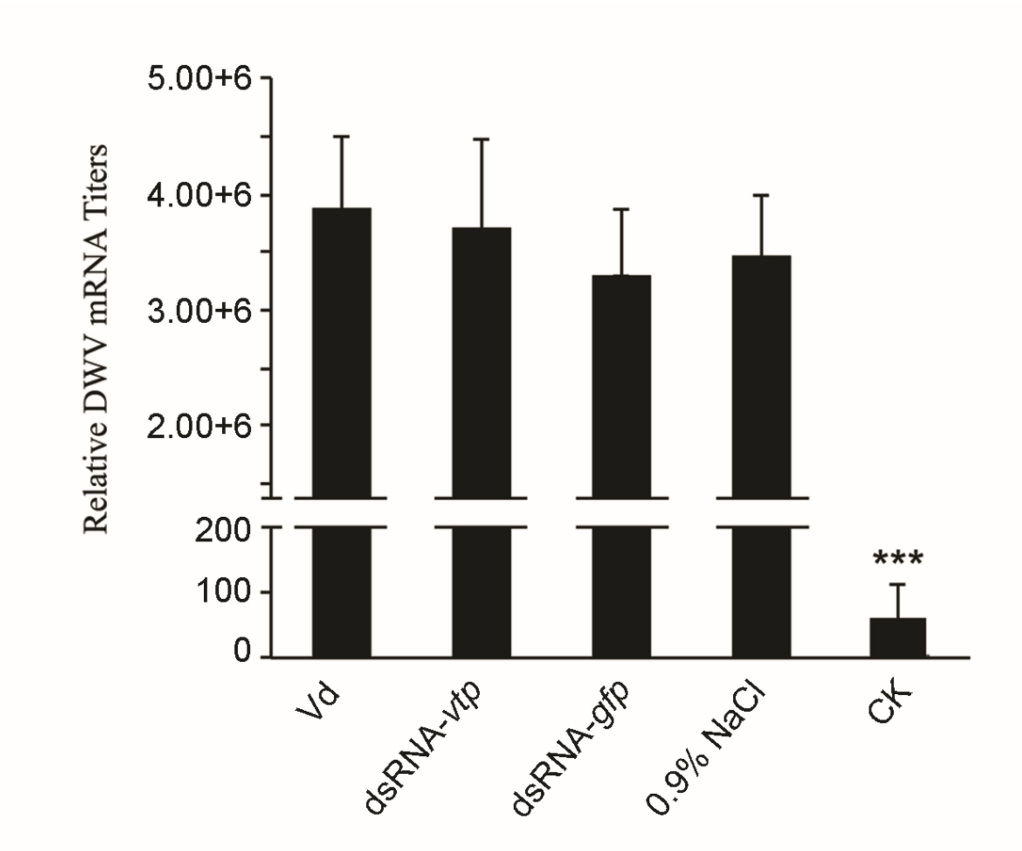

Supplementary Figure S5. Relative DWV mRNA levels in newly emerged worker bees (*A. mellifera*) infected by RNAi-treated *Varroa* mites. *A. mellifera* were infected with mites treated by overnight (approximately 15 hours) immersion in different solutions at 16°C. CK, No mites; Vd, *V. destructor* mites that were not treated; 0.9% NaCl, mites treated with 0.9% NaCl; dsRNA-*gfp*, mites treated with dsRNA-*gfp* (2.5 μg/μL); dsRNA-*vtp*, mites treated with dsRNA-*vtp* (2.5 μg/μL).

Supplementary Table S1. Quantitative titers of DWV in *A. cerana* adults developed from worker L5 larvae.

Treatment	Ct (Average ± SD)	ANOVA (df=8,35; F=0.441; P=0.885)
CK	34.1 ± 0.5	a
PBS	33.3 ± 1.1	a
TAG (2.0 ng/μL)	33.9 ± 0.5	a
E-VTP (0.01 ng/μL)	33.6 ± 0.8	a
E-VTP (0.1 ng/μL)	33.4 ± 1.6	a
E-VTP (1.0 ng/μL)	33.4 ± 1.8	a
E-VTP (2.0 ng/μL)	34.1 ± 0.8	a
E-VTP (5.0 ng/μL)	33.8 ± 0.5	a
NTC	33.2 ± 1.0	a

All the bee samples were collected and the DWV genome equivalents were measured by using qRT-PCR [34, 35]. CK, the adults developed from worker L5 larvae without any injection; PBS, the adults developed from worker L5 larvae injected with PBS (10 mM, pH 7.4); TAG, the adults developed from worker L5 larvae injected with purified tag protein from *E. coli* Transetta pET32(a). E-VTP (0.01 ng/μL, 0.1 ng/μL, 1.0 ng/μL, 2.0 ng/μL, and 5.0 ng/μL), the adults developed from worker L5 larvae injected with the purified E-VTP at different concentrations (0.01 ng/μL, 0.1 ng/μL, 1.0 ng/μL, 2.0 ng/μL, and 5.0 ng/μL). NTC: no template control during the qRT-PCR. Three replicates were established for each treatment. The data were analyzed using a normal one-way analysis of variance (ANOVA) using SPSS statistical software (16.0), and the significance of the between treatment differences was evaluated using Duncan's multiple range test, with significance indicated by a P value of < 0.05. The data presented are the mean values ± S.D. The same letters indicated no significant difference.

Supplementary Table S2. Primers used for the detection of viruses and microsporidia parasites.

Organism	Primer sequences	References
Deformed Wing Virus (DWV)	DWV-F: GACAAAATGACGAGGAGATTGTT<br>DWV-R: CAACTACCTGTAATGTCGTCGTGTT	5
Acute Paralysis Bee Virus (APBV)	ABPV-F: TTATGTGTCCAGAGACTGTATCCA<br>ABPV-R: GCTCCTATTGCTCGGTTTTTCGGT	31
Chronic Bee Paralysis Virus (CBPV)	CBPV-F: AGTTGTCATGGTTAACAGGATACGAG<br>CBPV-R: TCTAATCTTAGCACGAAAGCCGAG	31
Black Queen Cell Virus (BQCV)	BQCV-F: TGGTCAGCTCCCACTACCTTAAAC<br>BQCV-R: GCAACAAGAAGAAACGTAAACCAC	31
Kashmir Bee Virus (KBV)	KBV-F: GATGAACGTCGACCTATTGA<br>KBV-R: TGTGGGTTGGCTATGAGTCA	31
Sacbrood Virus (SBV)	SBV-F: GCTGAGGTAGGATCTTTGCGT<br>SBV-R: TCATCATCTTCACCATCCGA	31
Apis mellifera Filamentous Virus (AmFV)	AmFV -F: CAGAGAATTCGGTTTTTGTGAGTG<br>AmFV -R: CATGGTGGCCAAGTCTTGCT	40
Big Sioux River Virus (BSRV)	BSRV-F: RGTGCAGCTTTATGCGTTGCC<br>BSRV-R: CCGCTGTTGAGAATAAGGATATCCAGG	41
Lake Sinai Virus complex (LSV)	LSVdeg-F: GCCWCGRYTGTTGGTYCCCCC<br>LSVdeg-R: GAGGTGGCGGCGCSAGATAAAGT	42
Slow Bee Paralysis Virus (SBPV)	SBPV-F: GATTTGCGGAATCGTAATATTGTTTG<br>SBPV-R: ACCAGTTAGTACACTCCTGGTAACTTCG	43
Varroa destructor Macula-like virus (VdMLV)	VdMLV-F：ATCCCTTTTCAGTTCGCT<br>VdMLV-R：AGAAGAGACTTCAAGGAC	44
Israeli Acute Paralysis Virus (IAPV)	IAPV-F: AGACACCAATCACGGACCTCAC<br>IAPV-R: AGATTTGTCTGTCTCCCAGTGCACAT	45
Cloudy Wing Virus（CWV)	CWV-F: ATCAGCGCTTAGTGGAGGAA<br>CWV-R: TCGACAATTTTCGGACATCA	46
Apis Iridescent Virus（AIV）	AIV-F: GGCTAGTAAACGTAGTGGATATGACAAT<br>AIV-R: CACCTGGTGGTCCAAGAGAAG	47
Varroa destructor Virus 1 (VDV-1)	VDV1-R: CTTCCAAGGGCTCATCCATA<br>VDV1-F: CATGGAAATGGGATCAAACC	48
Moku Virus (MV)	MV-F: GTGCGATAGCTAAGCCTGAGATGG<br>MV-R: CAGTGCCCCCTATAGGTGTTGTT	49
Nosema.apis	*N.apis*-F: CCATTGCCGGATAAGAGAGT<br>*N.apis*-R: CCACCAAAAACTCCCAAGAG	32

Nosema.ceranae	*N.ceranae*-F: GACAACAAGGAAGACCTGGAAGTG<br>*N.ceranae*-R: TGTGAATAAGAGGGTGATCCTGTTGAG	32
